# First case report of post-operative infection due to *Francisella tularensis* after cardiac surgery

**DOI:** 10.1099/acmi.0.000035

**Published:** 2019-06-14

**Authors:** Arif Maqsood Ali, Muhammad Noor ul Amin, Shazia Arif

**Affiliations:** ^1^ Department of Pathology and Blood Bank, Rawalpindi Institute of Cardiology, Rawalpindi, Punjab, Pakistan; ^2^ Medical, AMC, Rawalpindi, Punjab, Pakistan

**Keywords:** *Francisella tularensis*, Zoonosis, infection, bacterium

## Abstract

**Background:**

*
Francisella tularensis* is a rare zoonotic bacterium that spreads sporadically by various routes, including infected arthropod bites, ingestion of contaminated water and inhalation of contaminated dust. However, its occurrence in postoperative chest infection has never been reported. Pathogen isolation, serology and molecular detection methods are commonly used for the diagnosis of tularaemia.

**Case presentation:**

We present the first case report of the isolation of *F. tularensis* from a patient with a chest infection (a boy in his teens) following cardiac surgery for closure of a ventral septal defect. It was isolated on blood and chocolate agar on the third day after the subculture of drain fluid collected in a blood culture bottle incubated in Bact T/Alert 3-D (bioMerieux, France). The organism was identified as *F. tularensis* by Vitek GN ID Cards (Vitek 2 Compact, bioMerieux, France). The patient made a smooth recovery with antibiotic therapy.

**Conclusion:**

*
F. tularensis* can cause post-operative infection, especially in patients with a rural background.

## Introduction

Tularaemia is a bacterial zoonotic disease caused by *Francisella tularensis *, which is a Gram-negative coccobacillus. The organism is thought to be maintained in the environment principally by various terrestrial and aquatic mammals, such as ground squirrels, rabbits, hares, voles, muskrats, water rats and other rodents [1]. It is highly infectious and may be transmitted to humans by a number of different routes. It is commonly spread by handling infected animals, ingestion of contaminated food or water, inhalation of infective aerosols, and bites from ticks and insects [2]. *F. tularensis* has three biovars: biovar A (biovar tularensis), which is highly virulent and is mostly found in North America and exceptionally in Europe [3]; biovar B, which is not as virulent as biovar A and has been traced in Europe, Asia and (rarely) North America; and biovar novicida, which was identified recently [3, 4].

Very few classical virulence factors have been identified for this pathogen. The bacterial capsule is not required for survival after phagocytosis of bacteria by polymorphonuclear leukocytes [5]. The lipopolysaccharide (LPS) of *F. tularensis* does not have the properties of a classical endotoxin. Moreover, it does not induce the release of interleukin-1 from mononuclear cells [6]. Phase variation of LPS and lipid A in *F. tularensis* has been observed. Probing the genome sequence data will help to identify putative virulence factors, such as adhesions [7].

The American Society for Microbiology has recommended biosafety level (BSL) 2 practices for dealing with clinically suspected specimens from patients of *F. tularensis* [8]. The diagnostic methods that are currently used in the developed world include pathogen isolation, molecular confirmation and serology [9]. Since culture procedures pose a risk to laboratory staff, serological tests can be carried out on nonvaccinated laboratory staff [10]. Antibodies against *F. tularensis* appear about 2 weeks after the onset of the disease. These can be detected in serum by either agglutination or by enzyme-linked immunosorbent assay (ELISA) [11]. Direct fluorescent antibody (DFA) assays can be carried in a reference laboratory to confirm the identification of the isolate as *F. tularensis* [12]. PCR-based methods have been evaluated for their potential to identify *F. tularensis* and to discriminate between its different subspecies. However, its highly infectious nature and the lack of a standardized PCR protocol makes the diagnosis difficult to confirm [13]. Real-time PCR assays have also been developed for the classification of *F. tularensis* subspecies [9]. Long random-sequence oligonucleotide primers and primers specific for repetitive extragenic palindromic (REP) sequences and enterobacterial repetitive intragenic consensus (ERIC) sequences are also being studied [14].

*F. tularensis* has been reported in humans and animals in every state of the USA except Hawaii [8]. Little is known about the distribution of *F. tularensis* in Asia except that it is present in the former USSR, eastern Turkey, China and Japan [15]. It was first reported in India in 2015 in blood cultures of a febrile patient [16]. The first case of tularaemia infection in humans was reported in 1980 in Marivan, in the Kurdistan Province of Iran [15]. However, there is a scarcity of data concerning *F. tularensis* in Pakistan. The only available study was carried out on *F. tularensis *-contaminated soil using PCR with the support of Pennsylvania State University, USA [17].

We did not find any report of *F. tularensis* being identified in clinical specimens from Pakistan [17]. We present the first case report of chest infection due to *F. tularensis *, which led to pleural and pericardial effusion in a cardiac patient who had been operated on for closure of a ventricular septal defect (VSD).

### Patient introduction

**Fig. 1.**
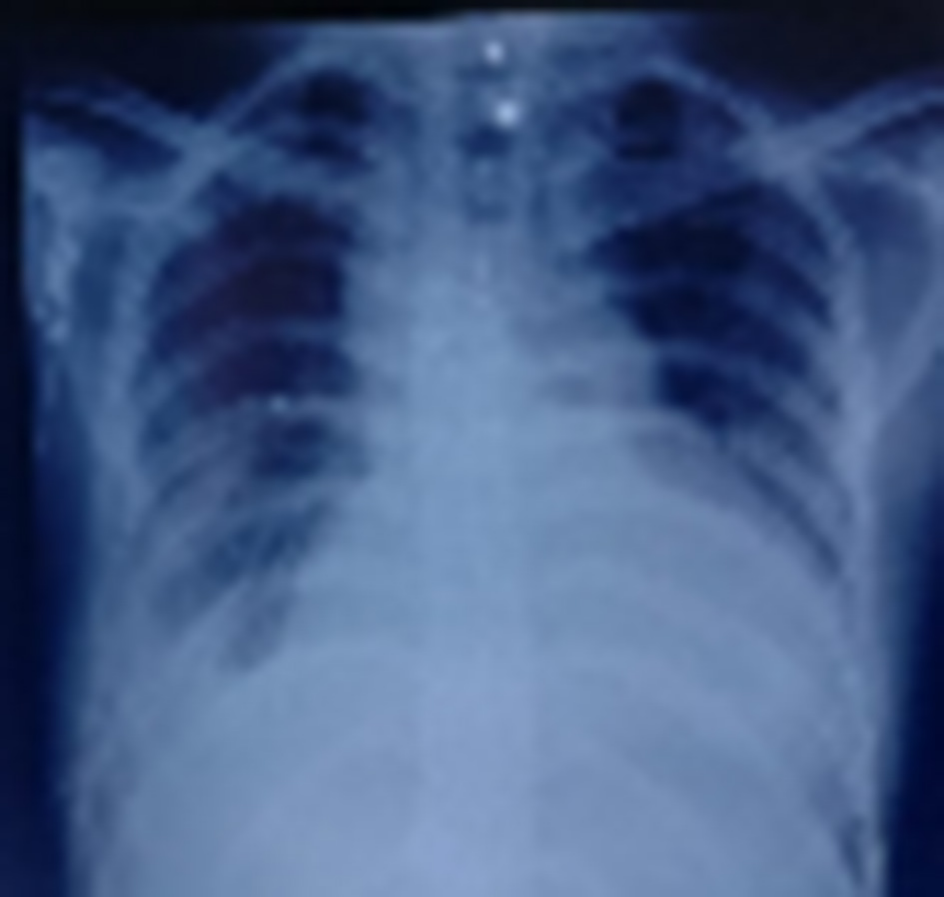
Chest X-ray showing right-sided pleural effusion with a chest tube and globular cardiac silhouette due to pericardial effusion.

A boy in his teens who had been diagnosed with VSD was operated on to effect its closure at Rawalpindi Institute of Cardiology (RIC), Rawalpindi, Pakistan in 2018. The patient was a resident of a rural area of Gujar Khan in Rawalpindi district in the province of Punjab, Pakistan. He had a history of close contact with birds at home, and cattle and farm animals in his neighbourhood. Well water was used for both bathing and drinking. The well was uncovered and did not have a surrounding parapet. The patient was discharged 6 days after surgery. However, the patient presented again to RIC 2 weeks later with fever and fullness in the right side of his chest. The wound was found to be infected with reddish exudate and was debrided. Chest X-rays showed right-sided pleural and pericardial effusion (Fig. 1). The pleural fluid was drained and found to be haemorrhagic, and its culture sensitivity was advised on. Laboratory investigations revealed a white blood cell count (WBC) of 15×10^9^ l^−1^ with 75 % polymorphs, a haemoglobin (Hb) level of 9.1 g dl^−1^ and a platelet level of 179×10^9^ l^−1^. The C-reactive protein (CRP) level was 36 g dl^−1^ and the erythrocyte sedimentation rate (ESR) 46 mm fell at the end of the first hour. The patient’s liver, renal and coagulation profiles were within normal limits. The serological test for *Brucella* antibodies was negative. A culture swab taken from the operation site did not yield any growth. The patient was treated with injections of ceftiaxone (1 g twice daily). However, the fever did not settle even after 5 days, although the chest wound was healing and granulating and the blood cultures were negative to date.

## Methods

The drain fluid from the right-sided pleural space was inoculated in Bact T/Alert 3-D (bioMerieux, France). After 4 days of incubation at 37 °C, the system showed a positive signal. Fluid was sub-cultured on blood and chocolate agar, and MacConkey agar. Biochemical tests such as a catalase test, an oxidase test, growth on urea and motility were performed using standard microbiological methods [18].

The saline suspension of the isolate was prepared to obtain a microbial concentration of 10^8^ colony-forming units (c.f.u.) ml^−1^. The bacterial suspension was inoculated in Vitek GN ID Cards and was identified by the Vitek-2 Compact (bioMerieux, France) [19, 20].

The isolate and its subsequent subcultures were identified using Vitek GN ID Cards in the Vitek-2 Compact (bioMerieux, France). The antibiotic susceptibility of the isolate was determined as recommended in the Clinical and Laboratory Standards Institute (CLSI) guidelines (M45A2) [21].

## Results

**Fig. 2.**
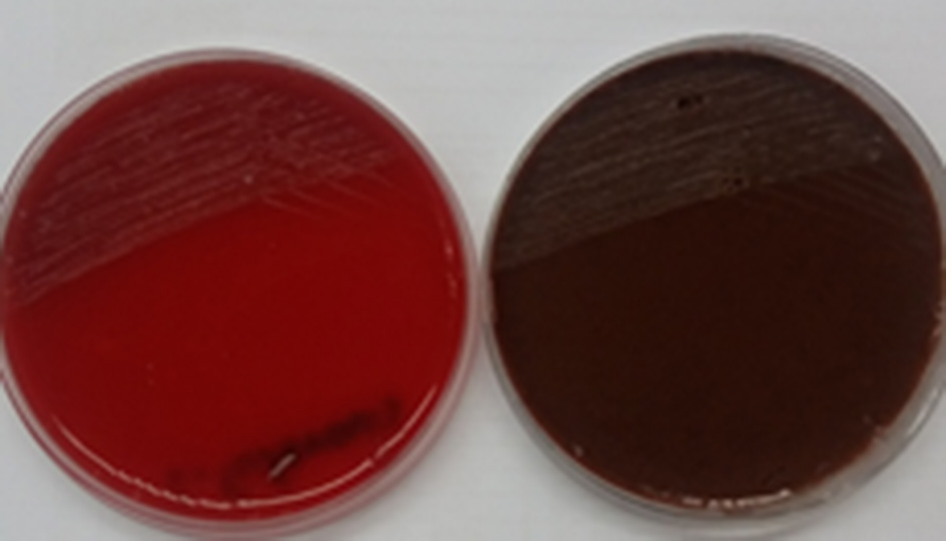
Growth of *F. tularensis* on blood and chocolate agar.

After 48 h of incubation at 37 °C, the subcultures showed a lawn of small non-haemolytic greyish white colonies with more than 10^5^ c.f.u. ml^−1^ on the blood and chocolate agar (Fig. 2). However, no growth was found on the MacConkey agar plate.

**Fig. 3.**
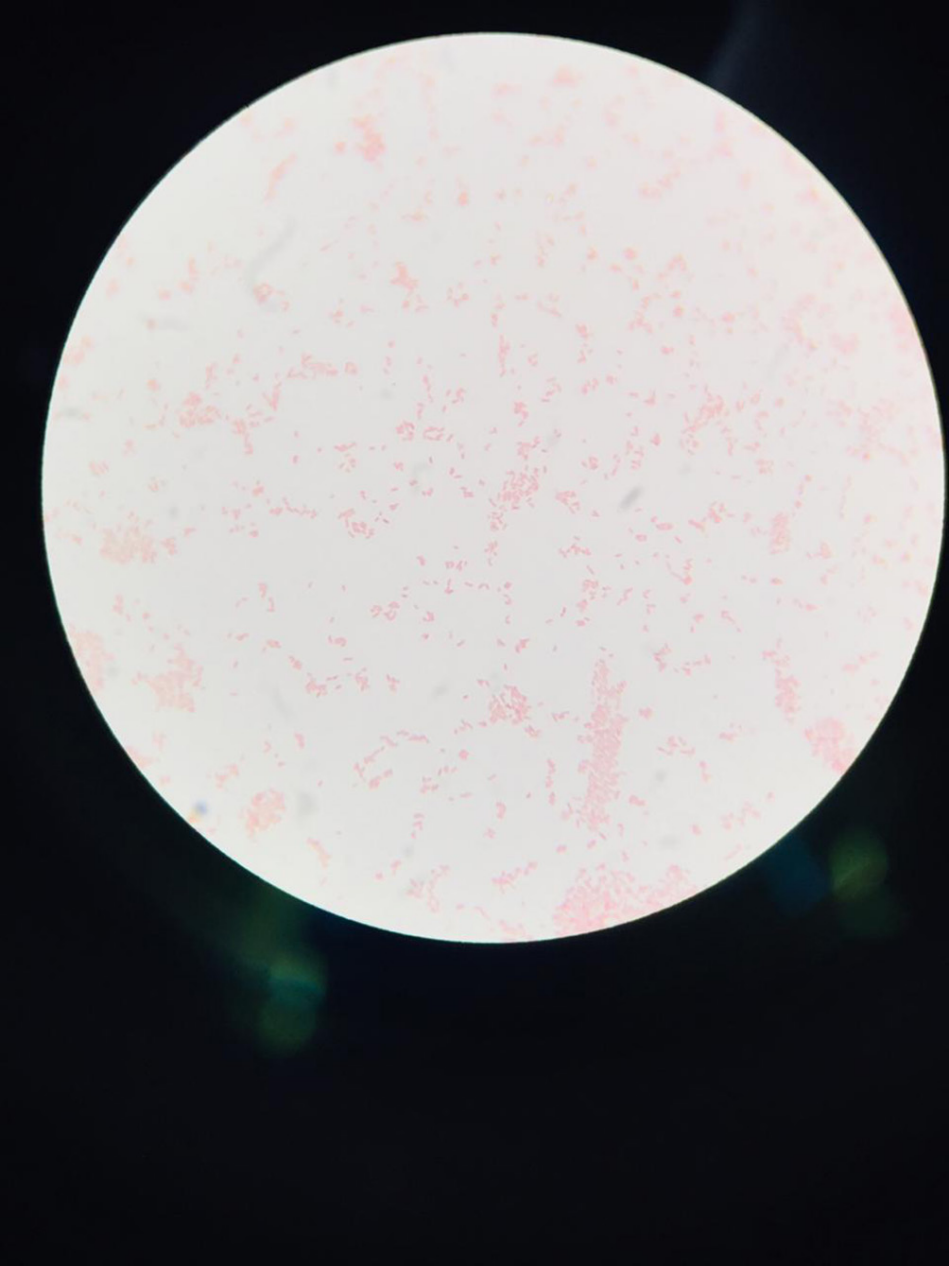
Gram staining of *F. tularensis *.

**Fig. 4.**
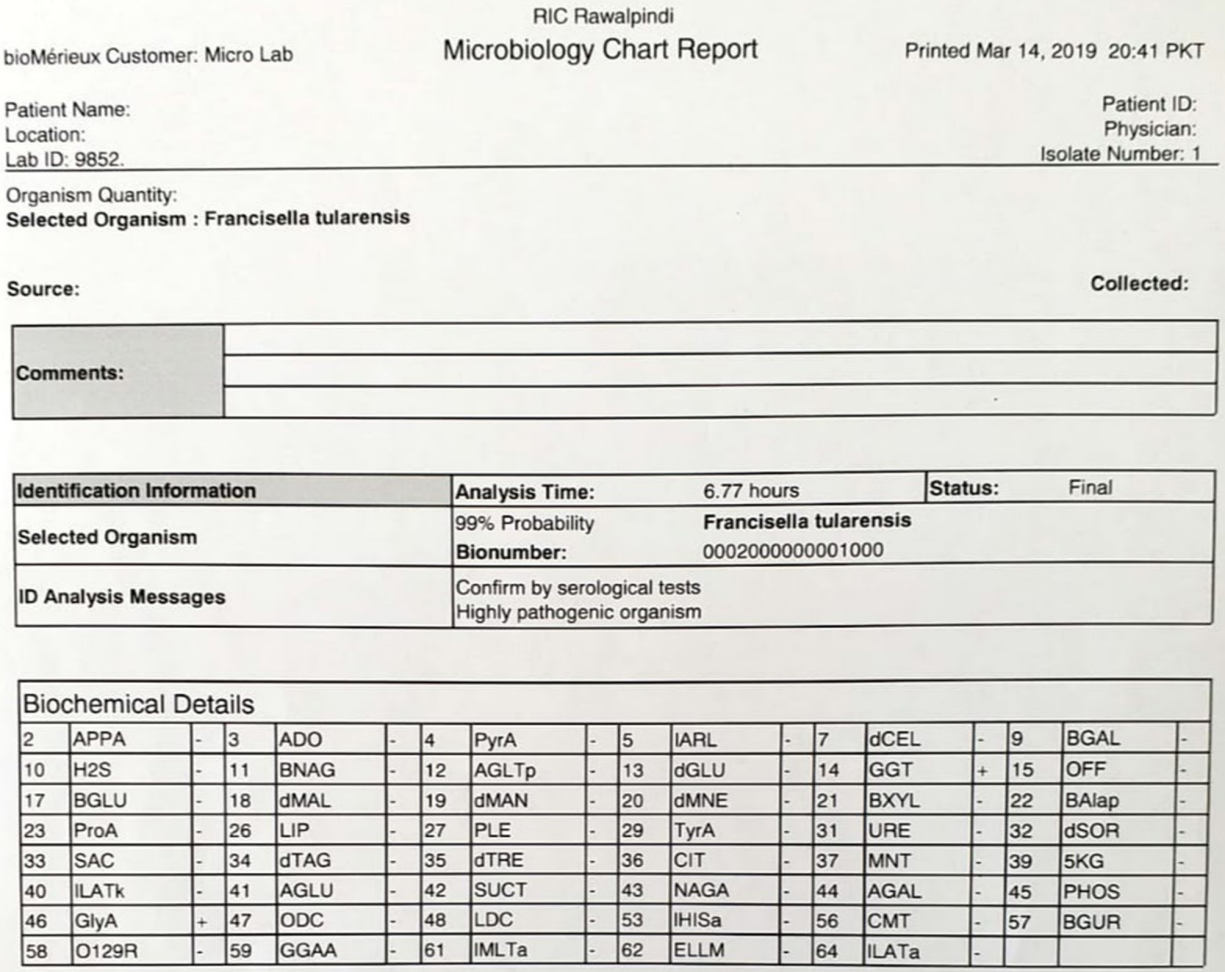
Vitek 2 Compact identification print-out from 14 March 2019.

**Fig. 5.**
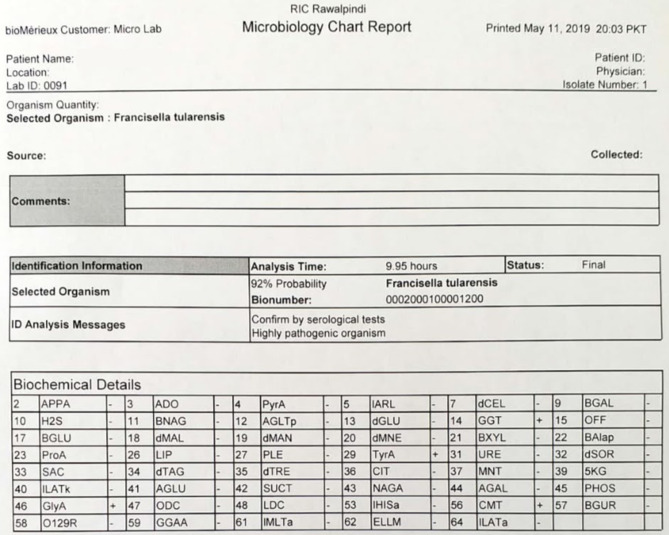
Vitek 2 Compact identification print-out from 11 May 2019.

Gram staining of the colonies showed small, faintly staining, Gram-negative cocobacilli (Fig. 3). The biochemical characteristics of the culture isolate are shown (Table 1). The organism was identified by Vitek 2 Compact as *F. tularensis* (Figs 4 and 5). It was susceptible to gentamycin, tobramycin, tetracycline and ciprofloxacin. Intravenous treatment with tobramycin was initiated and the patient became afebrile in the next 24 h. The patient improved dramatically after receiving antibiotics.

**Table 1.** Biochemical characteristics of the culture isolate

Gram stain	Catalase test	Oxidase test	Glucose	Growth in urea	Motility
Weakly staining Gram-negative cocobacilli	Negative	Negative	Fermented	Negative	Negative

### Outcome

**Fig. 6.**
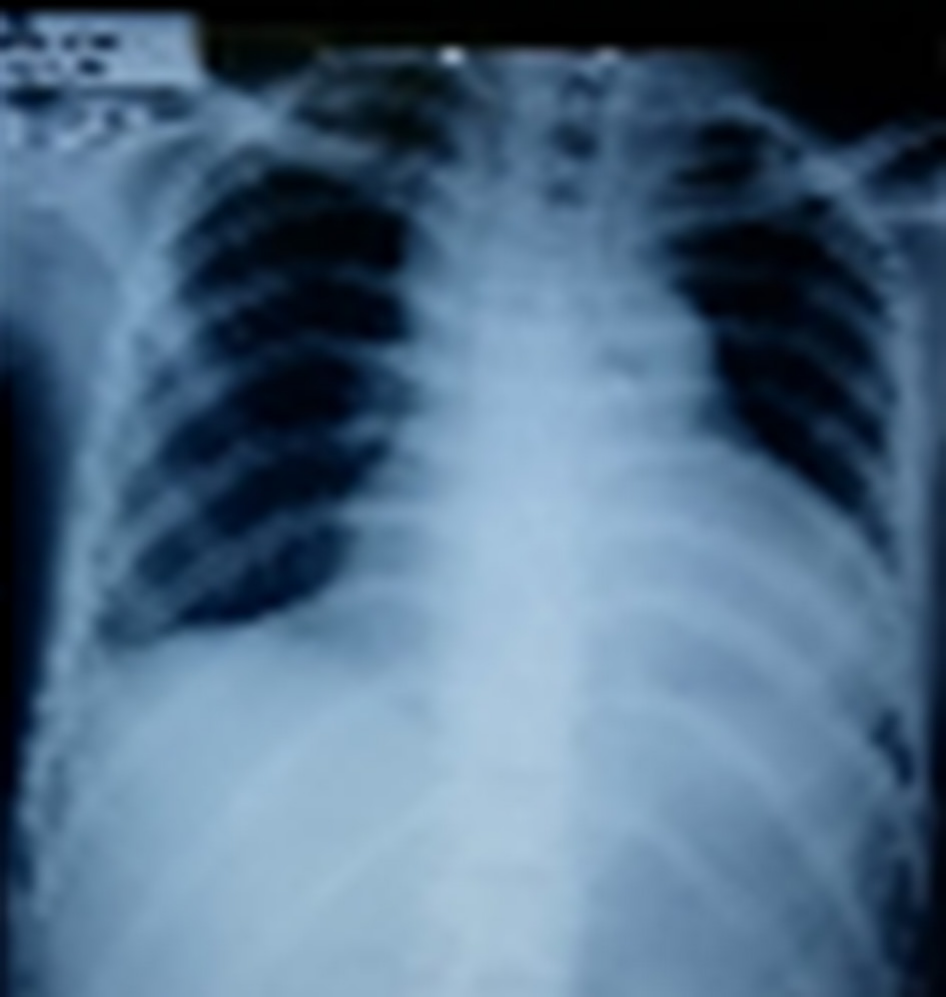
Chest X-rays showing resolution of pleural effusion.

The patient improved clinically. There was resolution of the chest infection and pleural effusion in the follow-up chest X-rays (Fig. 6). The other haematological and biochemical profiles also became normal. The chest tube was removed as the drain became dry. The patient was discharged in good condition after 10 days of treatment.

## Discussion

Most infections with *F. tularensis* have been reported from North America, Europe and northern Asia [8–12]. We report a novel and contemporary case of a post-operative chest infection with *F. tularensis* in a boy operated on for treatment of VSD. To the best of our knowledge, this is the first report of *F. tularensis* isolation in Pakistan and the first case of post-cardiac surgery chest infection due to the organism worldwide.

*F. tularensis* is a Gram-negative coccobacillus that causes tularaemia, a zoonotic disease primarily observed in the Northern Hemisphere. It is acquired by direct contact with infected animals or following the ingestion of contaminated water and food or the inhalation of contaminated aerosols [2]. The clinical presentation of the disease depends on the route of transmission. Symptoms develop within 3–5 days of infection and include fever with chills, sore throat, headache and generalized body aches. The clinical significance of tularaemia is unknown, but it can be fatal in patients with other comorbid illnesses, such as diabetes mellitus and pneumonia [9]. Our patient was operated on for VSD closure but developed a post-operative chest infection with right-sided pleural effusion 2 weeks after discharge from the hospital (Fig. 1). He had a history of consumption of open well water for bathing purposes only. Cattle and farm animals in his neighborhood with associated tick, deer fly or mosquito bites are another possible route of transmission. The source of infection in our patient is probably environmental in origin.

The first case of *F. tularensis* from India was reported in a febrile patient with positive blood culture that was similarly identified by Vitek-2 [16]. A rare case report of pneumonia due *to F. tularensis* was reported from Turkey in an elderly lady with a history of contact with infected animals [22]. There were also both birds and farm animals in our patient’s neighborhood.

The organism is rarely isolated from blood culture directly, but has been identified with the development of sensitive blood culture systems [23]. Our patient also had bloody pleural fluid that yielded greyish white non-haemolytic colonies on blood and chocolate agar, with no growth on MacConkey agar. These were negative for oxidase, urease, motility and fermentation of lactose and other sugars. Similar findings have been reported in a study from Sudan, where *F. tularensis* was isolated from blood culture in brain heart infusion [24]. A similar case of *F. tularensis* isolation has been reported from haemorrhagic pleural effusion and blood specimen in a middle aged man from Norway with sepsis [25].

*F. tularensis* is known as a potential bioterrorism agent due to its high virulence and low infective dose [23]. Tularaemia is the most commonly reported laboratory-acquired bacterial infection. Culture techniques are usually avoided for diagnosis because of the high virulence of *F. tularensis* infections, especially in areas where biovar A is highly prevalent [9].

*F. tularensis* is a fastidious aerobic organism that is usually slow to grow and difficult to culture due to its requirement for cysteine for enhanced growth [23]. In our patient the drain fluid was inoculated in blood culture bottles and incubated in Bact T/Alert 3-D (bioMerieux, France) that showed signal positivity on the fourth day. The fluid from signal-positive culture bottles showed minimal growth of small, non-haemolytic, greyish white colonies on subculture after 48 h on chocolate and blood agar (Fig. 2). Gram staining of the colonies showed small, faintly staining, Gram-negative cocobacilli. (Fig. 3). *F. tularensis* is relatively inert biochemically, with only a few sugars (glucose, maltose, sucrose and glycerol) being utilized. *F. tularensis* does not reduce nitrate. It is oxidase- and urease-negative and is weakly catalase-positive [15, 18].

The phenotypic and biochemical characteristics of our patient’s isolate were characteristic for *F. tularensis* (Table 1). The isolate and its subsequent subcultures were identified by Vitek-2 as *F. tularensis* (Figs 4 and 5).

Serological-based tests can be used in setups that lack the recommended biosafety level facilities for bacterial culture. The detection of serum antibodies is most frequently achieved by agglutination or by ELISA. An immunochromatographic handheld assay can detect polyclonal and a monoclonal antibody to the LPS of *F. tularensis* [23]. However, these tests are not readily available in our country.

Fluorescence-labelled antibodies can be used to detect *F. tularensis* in clinical specimens. PCR-based assays can be used to identify *F. tularensis* DNA in human skin lesions or in the blood of infected mice [12]. These procedures permit a more rapid diagnosis, but are usually performed in reference laboratories. Only one PCR-based study on *F. tularensis *-contaminated soil has been reported from Pakistan, and no study on human infection has so far been reported [17]. Long random-sequence oligonucleotide primers and primers specific for REP and ERIC sequences are also being evaluated in the developed world [23].

Thus, in most clinical laboratories a diagnosis of tularaemia relies on serological tests, except in patients suspected of tularaemia with possible bacteraemia, where blood cultures can yield *F.* *tularensis* [5, 6]. In our country, no study has been carried out to isolate *F. tularensis* from clinical specimens [17]. This is either due to the rarity of the disease or because there is often a lack of diagnostic facilities in our country. Confirmation of the isolate with antigen detection or molecular methods could not be performed due to the lack of these facilities in our country and due to failure to transport it, since the organism is a bioterrorism agent.

### Conclusion

We report the first case of *F. tularensis* isolated from the haemorrhagic pleural fluid of a post-cardiac surgery patient from a rural background in Pakistan. The specimens in clinically suspected cases can yield *F. tularensis* on enriched media on subculture from infected fluid. Moreover, there is a dire need for diagnostic facilities to identify the pathogen in our country for early detection and treatment of clinically suspected patients. Until that time vigilance is required by clinicians and microbiologists to identify suspected cases.

## Data bibliography
